# Experiences of healthcare encounters among patients using complementary and alternative medicine alongside oncology or palliative care: a qualitative interview study in Sweden

**DOI:** 10.1136/bmjopen-2026-123561

**Published:** 2026-09-18

**Authors:** Mikael Källman, Georg Holgersson, Stefan Bergström, Lisa Martinsson, Britt-Marie Lindgren, Michael Bergqvist

**Affiliations:** 1 Department of Diagnostics and Intervention, Umeå University, Umeå, Sweden; 2 Centre for Research and Development, Uppsala University/Region Gävleborg, Umeå, Sweden; 3 Department of Oncology, Gävle Hospital, Umeå, Sweden; 4 Department of Oncology, Uppsala Academic Hospital, Uppsala, Sweden; 5 Umeå University Department of Diagnostics and Intervention, Umeå, Sweden; 6 Nursing, Umea Universitet, Umeå, Sweden

**Keywords:** Cancer, ONCOLOGY, Patient-Centered Care, Patient Preference, COMPLEMENTARY MEDICINE

## Abstract

**Abstract:**

**Objectives:**

A substantial proportion of patients with cancer use complementary and alternative medicine (CAM), yet the topic is rarely addressed in clinical encounters. This communication gap may affect patient safety, trust and participation in care. This study aimed to explore how patients experience encounters with healthcare professionals when using CAM alongside oncological or palliative care.

**Design:**

An exploratory qualitative study using semi-structured interviews and qualitative content analysis.

**Setting:**

Oncology and palliative care services in a Swedish healthcare region.

**Participants:**

19 adult patients with cancer who used CAM alongside conventional cancer care and were purposively recruited from a larger regional survey.

**Results:**

Patients described continuously assessing whether it was appropriate to mention CAM. Subtle verbal and non-verbal cues from healthcare professionals shaped perceptions of what was considered legitimate within clinical encounters, often leading to silence or withdrawal. In contrast, neutral or respectful responses facilitated trust and dialogue. Openness regarding CAM was not fixed but adjusted from one encounter to another, influenced by interpersonal interactions, organisational conditions and prevailing evidence norms. Participants expressed a need for respectful dialogue, basic competence among healthcare staff and practical support for safe CAM use, without implying endorsement.

**Conclusions:**

Patients who use CAM navigate a healthcare context characterised by shifting relational cues and structural constraints that influence communication. Safe, person-centred care may be supported by normalising discussions about CAM, improving basic staff knowledge and facilitating open dialogue. Such measures may promote trust, improve communication and reduce risks associated with undisclosed CAM use.

**STRENGTHS AND LIMITATIONS OF THIS STUDY**

- Qualitative interviews enabled in-depth exploration of patients’ experiences.
- Inclusion of both oncology and palliative care contexts broadened perspectives.
- Conducted within one Swedish region, which may limit transferability.
- Participants were complementary and alternative medicine users willing to be interviewed; other perspectives may be underrepresented.
- Findings are based on retrospective accounts and should be understood as contextual and interpretive.

## Background

 According to the WHO, the use of traditional, complementary and integrative medicine is widespread among people living with cancer, and many patients use such approaches alongside conventional oncological or palliative care.^1^

Swedish survey data indicate that approximately one-third of patients use complementary and alternative medicine (CAM) after a cancer diagnosis, with lifetime use rates even higher; however, most do not discuss CAM with physicians and nurses. These patterns are consistent with international findings and highlight a persistent communication gap in routine care.^2 3^ Integrative oncology has emerged as one response to this gap. It incorporates mind–body practices, natural products and lifestyle interventions alongside conventional treatment to optimise quality of life, ease symptoms and support participation across the cancer trajectory.^4 5^

Guidance has evolved accordingly: joint recommendations from the Society for Integrative Oncology and the American Society of Clinical Oncology (SIO–ASCO) now provide evidence-informed advice for selected modalities—such as acupuncture, massage, yoga, hypnosis and other mind–body interventions—particularly for cancer-related pain, anxiety/depression and fatigue, emphasising non-pharmacological options while also acknowledging implementation barriers.^6 7^

Patient-centred care provides a broader foundation for these advancements. In integrative oncology, patient-centredness is defined as care that is evidence-informed, grounded in patients’ values and preferences and responsive to their lived experiences across the cancer trajectory. For patients who use CAM, feeling that the topic is legitimate—and being met with curiosity rather than judgement—is closely tied to trust, safety and a sense of participation in care.^5^ Despite this need, discussions about CAM are not routine in clinical practice.

Patients frequently wait for healthcare staff to initiate the topic, while clinician-initiated inquiries about CAM use are uncommon, as shown in international reviews as well as recent European studies.^8 9^

Neutral conversations foster confidence, whereas restrictive tones can lead to patient withdrawal and overlooked opportunities for supportive care.^9 10^

Our research conducted in Sweden aligns with these trends. In a regional survey, 34% reported CAM use after diagnosis and ~70% of users had not discussed this with healthcare staff; many nevertheless wished that certain CAM options were addressed in or co-ordinated with conventional care. Earlier Swedish research similarly shows low disclosure despite expressed interest in professional guidance. Collectively, these findings suggest a need to normalise brief, neutral invitations to talk about CAM, supported by basic staff competence and clear signposting to reliable evidence resources.^2 3^ In this context, an integrative, person-centred approach does not equate to endorsing or providing CAM. Rather, it acknowledges that CAM is part of many patients lived experiences and that effective communication regarding possible benefits, uncertainties, risks and interactions constitutes good clinical practice. This perspective is in accordance with current SIO–ASCO guidance and current discussions about implementation within oncology.^6 11^

Previous research has largely focused on prevalence, disclosure rates and clinicians’ perspectives, whereas patients’ lived experiences of engaging in discussions about CAM with healthcare staff have received limited attention.^8 12^ Given this knowledge gap, an exploratory qualitative approach was considered appropriate.

The aim of this study was to illuminate how patients with cancer who use CAM alongside oncological or palliative care experience encounters with healthcare staff.

## Methods

### Study design

This study had a qualitative design using semi-structured interviews. Data were analysed using qualitative content analysis according to Graneheim and co-workers.^13–15^

### Context

This study is part of a larger research project investigating the use and perceptions of CAM among patients with cancer.^2 16^ The present study constitutes a qualitative sub-study based on participants recruited from a survey conducted among patients receiving cancer treatment in the Gävleborg region.

### Sampling and participant selection

Participants were recruited from a larger survey conducted among patients receiving adjuvant chemotherapy or palliative home care at one hospital in the south of North/Mid Sweden.^2 16^

Eligible participants were adults (≥18 years) receiving either adjuvant chemotherapy at the oncology department in Region Gävleborg or palliative cancer care through the regional palliative home-care service. Exclusion criteria included previous chemotherapy within the Region Gävleborg healthcare system, cognitive impairment or other comorbidities assessed by the clinical staff and research team as likely to interfere with the individual’s ability to provide informed consent or participate in the interview. No formal cognitive assessment was performed. Eligibility regarding cognitive capacity was determined through clinical judgement by the healthcare staff involved in the patients’ care.

**Figure 1:**
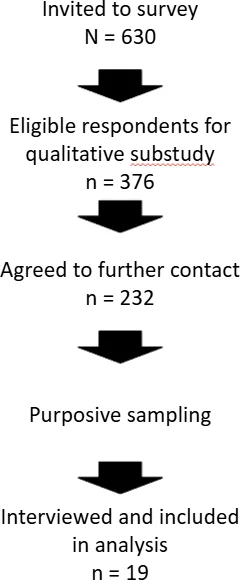
Flowchart of participant recruitment and sampling for the qualitative substudy. The figure illustrates the recruitment process from survey invitation to final inclusion of 19 interviewed participants.

Of the 630 patients invited to participate in the survey, 376 provided responses that met the eligibility criteria for the present substudy (fig 1). Among these respondents, 232 (62%) agreed to be contacted for a follow-up interview and provided their contact details.

A purposive sampling strategy was used to achieve variation in age, sex, treatment context (oncology or palliative care) and experiences of CAM use.

Only participants who had provided written consent to be contacted for further research and who provided verbal informed consent prior to the interview were included in this substudy.

Participants were recruited from both adjuvant oncology and palliative care settings to capture variation in experiences across different phases of the cancer trajectory. Although these groups differ in terms of treatment goals and clinical context, CAM use has been reported in both populations and communication with healthcare professionals regarding CAM may influence patients’ experiences of care.

### Data collection

Of the participants who completed the survey study, those who reported CAM use and consented to be contacted for further research were considered eligible for the qualitative substudy. Purposeful sampling was used to obtain variation in age, sex, cancer diagnosis and experiences of CAM use. Eligible participants were contacted and invited to participate in an interview, resulting in a final sample of 19 participants.

19 patients participated in semi-structured interviews which were conducted over a 2 year period. Participants had provided written informed consent in the initial survey and reconfirmed verbal consent prior to the interviews after receiving detailed study information. The interviews were conducted either face-to-face at care sites or via telephone, depending on participant preference.

An interview guide was developed by the research team based on domains included in the previous survey study.^2 15^ The guide was designed to explore participants’ experiences, motivations and perceptions regarding CAM use in the context of cancer care. Topics included experiences of CAM use, communication with healthcare professionals about CAM, perceived responses from healthcare staff, information needs and views on how healthcare services could address CAM-related questions. Open-ended questions addressed participants’ experiences of encounters with healthcare professionals in relation to CAM. The semi-structured format allowed participants to elaborate on their experiences and enabled the interviewer to ask follow-up questions while ensuring that key topics relevant to the study aim were covered. Follow-up questions such as ‘Can you tell me more about that?’, ‘How did that make you feel?’ and ‘What happened then?’ were used to encourage elaboration and generate rich descriptions. All interviews were conducted by the first author (MK), audio-recorded and transcribed verbatim. The total interview time was 608 min (mean 26 min; range 11–58 min).

### Data analysis

Data were analysed manually using qualitative content analysis, following the methodology described by Graneheim and co-workers.^13–15^ The interviews were read repeatedly to achieve a sense of the whole. Meaning units related to the study aim were identified, condensed and coded. Codes were then grouped into sub-categories and categories representing the manifest content. Through an interpretive analytic process, themes were developed to capture patterns of latent meaning across the data.

The primary analysis was conducted by one researcher (MK) using a structured and reflexive approach. Preliminary categories and themes were discussed with senior researchers (LM and B-ML) during analytic consultations to enhance credibility and reflexivity. Representative quotations were selected to illustrate how participants’ statements supported the categories and themes. The analysis followed the principles of qualitative content analysis as developed by Graneheim and co-workers, with attention to levels of abstraction and interpretation, maintaining participants’ voices as central, and avoiding causal interpretations in theme formulation. Reflexivity and transparency were emphasised to support trustworthiness.^14 15 17^

### Patient and public involvement

Patients and the public were not involved in the design, conduct, reporting or dissemination plans of this research.

## Results

The qualitative content analysis resulted in three themes, each comprising subthemes (table 1). Together, the findings describe how patients who use CAM navigate communication within oncology and palliative care, and how openness, trust and responsibility are shaped in everyday encounters with healthcare staff.

**Table 1:** Overview of subthemes and themes revealed during the analysis

Subtheme	Theme
Facing ambiguity and limited communication Facing scepticism and mistrust Facing deficient engagement and limited knowledge	Navigating a healthcare landscape shaped by varying responses, support and structures
Experiencing limited opportunities to share information Facing evidence requirements that constrain dialogue	Adjusting openness in encounters shaped by relational cues and normative expectations
Experiencing a need for dialogue grounded in respect and competence Experiencing the importance of continuity, safety and hope	Seeking healthcare that embraces dialogue about CAM with openness, competence and human presence

CAM, complementary and alternative medicine.

### Navigating a healthcare landscape shaped by varying responses, support and structures

Participants’ accounts revealed a continuous process of relational navigation in which they assessed when and how CAM could be mentioned. This navigation involved attentiveness to interpersonal cues, organisational conditions, and implicit boundaries regarding what was considered legitimate within healthcare encounters.

#### Facing ambiguity and limited communication

Participants described that healthcare staff seldom addressed CAM, even when other aspects of care functioned well. While some participants described this absence of inquiry as expected, others actively reflected on it as meaningful and consequential.

The lack of questions or information was not experienced as neutral, but as an implicit signal that such topics fell outside the scope of legitimate clinical dialogue. This silence contributed to uncertainty about what could be discussed and reinforced the sense that CAM was positioned at the margins of care.

I was really positive about the way I was treated in every way, but there was no talk or information about any alternative treatments or anything like that. (17)

No one ever asked.^13^

#### Facing scepticism and mistrust

When CAM was raised, participants described how sceptical or subtly dismissive responses from healthcare staff shaped both their understanding of professional boundaries and their own sense of trust. These reactions—often conveyed through tone of voice, brief comments, or non-verbal cues rather than explicit rejection—were interpreted as signals of what was considered acceptable within biomedical norms. In some cases, these responses included cautionary statements about potential harm, which were experienced as alarming or exaggerated. For example, one participant recalled being told that using CAM “could backfire—that I might get more tumours if I used that” (8), illustrating how such messages could be perceived as both dismissive and fear-inducing.

Experiences of being questioned, subtly rejected, or not taken seriously contributed to growing caution and mistrust, influencing how participants positioned themselves and often leading to withdrawal from further discussion in future encounters.

You can hear straight away it’s not accepted—it’s only their way that counts. (5)

They said it could backfire—that I might get more tumours if I used that. (8)

#### Facing deficient engagement and limited knowledge

The participants described how limited engagement and knowledge regarding CAM within healthcare encounters led them to assume responsibility for initiating dialogue, coordinating care and ensuring continuity.

Across accounts, participants described taking increasing responsibility for navigating care when engagement or knowledge about CAM was perceived as limited. This included initiating discussions, requesting investigations, following up results and coordinating between services. Over time, this contributed to feelings of being positioned at the margins of care rather than at its centre, particularly when organisational rules were seen to restrict staff engagement with CAM.

It feels like healthcare doesn’t drive anything—the only thing driving my care is me, and I feel that wherever I go. (6)

I feel like the Black Peter in healthcare—no doctor wants to take responsibility for me. (4)

### Adjusting openness in encounters shaped by relational cues and normative expectations

Openness about CAM was described as fluid rather than stable, shifting depending on whether the encounter felt relationally safe. Participants portrayed disclosure as something carefully adjusted from one encounter to another, depending on how receptive the context appeared to be.

#### Experiencing limited opportunities to share information

Participants described disclosure of CAM use as situational and relational rather than fixed. While some withheld information unless explicitly asked, others described selectively sharing depending on how healthcare professionals responded. Several participants described ‘feeling their way’ in conversations, testing receptiveness before continuing.

Silence or lack of inquiry was interpreted as an indication that CAM was not relevant or welcome, reinforcing a cautious and sometimes provisional approach to disclosure. In some cases, participants chose not to share at all, even when they did not consider their use of CAM to be problematic.

If they don’t ask, I don’t tell. (12)

I usually mention it, but you notice pretty quickly if it’s worth continuing. (3)

I haven’t told them—I don’t think they want to hear it. (1)

#### Facing evidence requirements that constrain dialogue

Participants also described how prevailing evidence norms shaped the limits of dialogue. When CAM did not align with what was considered clinically proven, discussions were often curtailed or avoided altogether.

These moments were experienced as conversational closures, signalling that certain perspectives did not fit within professional or institutional frameworks. While some participants perceived individual healthcare professionals as personally interested, this openness was often constrained by formal expectations of evidence.

They won’t talk about anything unless it’s clinically proven—then the door closes. (9)

They say they can’t discuss it because there isn’t enough evidence. (12)

### Seeking healthcare that embraces dialogue about CAM with openness, competence and human presence

Alongside descriptions of constraint, participants articulated clear wishes regarding healthcare’s role in relation to CAM. These wishes centred on relational openness, basic competence and practical conditions that could enable safer and more humane care.

#### Experiencing a need for dialogue grounded in respect and competence

Participants did not expect healthcare staff to be experts in CAM, but emphasised the importance of respectful dialogue and at least a basic level of knowledge. While some described moments of openness, many experienced that lack of competence limited opportunities for meaningful discussion.

In the absence of such competence, participants described feeling dismissed or marginalised, as if their experiences fell outside the scope of legitimate knowledge within healthcare.

They know nothing about alternatives—I wish they had some knowledge. (10)

I asked what they thought, but I didn’t get any real answers. (9)

#### Experiencing the importance of continuity, safety and hope

Beyond information, participants highlighted the importance of continuity, relational presence and emotional support. Being seen, listened to and taken seriously was described as fundamental for maintaining trust, particularly in the context of serious illness.

Participants also emphasised the importance of preserving hope. Encounters that were perceived as dismissive or overly focused on prognosis were experienced as undermining this, whereas even small expressions of encouragement could have a significant impact.

For some, this included a wish for safer and more integrated conditions for using CAM, while for others it reflected an existential dimension of care in which hope and meaning were protected.

You must never take away hope; that’s the last thing a person has. (9)

I want someone I can contact—someone who knows me. (4)

Taken together, participants described that engagement with CAM in healthcare did not necessarily require endorsement, but rather an openness to dialogue and a willingness to engage with their perspectives. Relational aspects, such as being met with openness and respect, were described as important for fostering trust and a sense of safety. At the same time, participants acknowledged that healthcare professionals operated within certain constraints, which could limit their ability to engage with CAM-related questions. These experiences highlighted the importance of having space for dialogue about CAM within clinical encounters.

## Discussion

This study highlights how conversations about CAM became either legitimate or illegitimate to raise in oncology and palliative care. Rather than reflecting fixed attitudes, openness appeared as something continually calibrated in relation to shifting permissions, subtle cues and professional boundaries. These dynamics were not only interpersonal; they were also shaped by broader expectations about evidence, risk and legitimacy within biomedical practice.

Participants described encounters that ranged from muted or dismissive responses to brief moments of curiosity and affirmation. This variation suggests that openness to CAM was dynamic and shaped by relational and contextual conditions within each encounter.

This pattern corresponds with prior work showing that nondisclosure of CAM is common and is often linked to anticipated disapproval, a lack of clinician inquiry or a perception that CAM falls outside routine oncological care.^12 18^ When the relational climate felt closed, individuals tended to protect rapport by withholding information; when responses were neutral or inviting, dialogue became possible. These observations are consistent with studies connecting patient-centred communication with greater disclosure of complementary approaches.^19^

A central insight in our study was the role of non-inquiry. Silence from clinicians was not experienced as neutral but as a boundary signal about what belonged in the consultation. The absence of questions thus shaped participants’ appraisal of what was permissible, reinforcing selective openness rather than spontaneous disclosure—an interpretation echoed earlier descriptions of the ‘they don’t ask, so I don’t tell’ dynamic in cancer care.^10 12^

Similar patterns have been synthesised in recent reviews, showing that non-inquiry and anticipated disapproval remain key reasons for CAM non-disclosure across oncology settings.^9^

Discussions frequently stalled when CAM did not align with prevailing evidence hierarchies. When modalities lacked conventional proof, the interactional space often narrowed, and participants perceived that their experiential knowledge carried limited legitimacy. This tension between evidence-based norms and patients’ values is well described and can heighten power asymmetries if not explicitly addressed.^20–22^ At the same time, guideline movements within integrative oncology illustrate that a selective, evidence-informed dialogue is both possible and desirable, particularly for symptom management.^6 23^

Recent research also illustrates the importance of maintaining an evidence-informed perspective within integrative oncology. For example, a randomised controlled trial among breast cancer survivors found that melatonin supplementation in combination with an exercise programme did not provide additional benefits for anthropometric outcomes, quality of life or hormonal responses beyond those achieved through exercise alone.^24^ These findings highlight the importance of evaluating complementary interventions using rigorous methodologies and support the need for balanced discussions about potential benefits, limitations and uncertainties related to CAM use in cancer care. In parallel, qualitative studies from challenging clinical contexts describe how evidence hierarchies and professional uncertainty can narrow dialogue and amplify power asymmetries between patients and clinicians.^22^

Organisational and professional structures further shaped communicative possibilities. Interviewees described perceived prohibitions, documentation demands and time constraints that restricted opportunities for nuanced dialogue—even when individual clinicians were curious. Such conditions align with previous findings suggesting that organisational factors, rather than individual attitudes alone, shape whether CAM can be meaningfully discussed. At the same time, studies indicate that brief, structured conversations about CAM can be integrated without compromising safety when supported by simple tools and trained facilitation.^25–29^ International research further highlights variability in how integrative oncology services are implemented, underscoring how local organisational conditions influence opportunities for dialogue.^30^

Beyond information exchange, participants in the present study emphasised the existential and relational dimensions of care, where being recognised and listened to fostered trust and participation in care. Previous research highlights that requests for continuity and humane engagement point to the importance of care that attends to the whole person, not only the disease—an orientation consistent with person-centred principles in integrative oncology.^5 6 11^ Taken together, these findings suggest that conversations about CAM function as a relational barometer in cancer care. Openness emerged when safety, legitimacy and recognition were perceived, whereas withdrawal occurred when implicit or explicit boundaries signalled that CAM was unwelcome. A pragmatic implication is to incorporate brief, neutral invitations to discuss CAM in routine consultations, alongside ensuring basic staff competence and providing access to trustworthy evidence resources.^6 7 23^ Such measures may reduce the risks associated with undisclosed CAM use while strengthening trust and supporting more person-centred care. Recent randomised trials of structured open dialogue about CAM show that such conversations do not automatically improve quality of life, underscoring the importance of careful, person-oriented and safety-focused implementation.^31^

Finally, these findings align with Swedish data demonstrating substantial use of CAM alongside limited discussion with clinicians—despite a clear desire for dialogue and, in some cases, for selected modalities to be acknowledged or integrated into conventional care. This illustrates a local practice gap that our material helps explain at the level of lived experience in oncology and palliative contexts.^2 3^

Although the present study was conducted within a Swedish healthcare context, the communication patterns described by participants appear consistent with findings reported internationally. Previous studies from diverse healthcare settings have similarly identified limited clinician inquiry about CAM, concerns about professional disapproval, selective disclosure, and challenges related to discussing CAM within evidence-based clinical frameworks. These similarities suggest that the communication barriers identified in the present study are unlikely to be unique to the Swedish healthcare system but may reflect broader challenges in oncology and palliative care internationally.^9 10 12 18^

Future research should evaluate educational interventions aimed at improving healthcare professionals’ competence and confidence in discussing CAM within oncology and palliative care. Such interventions could focus on communication skills, evidence-informed CAM knowledge and shared decision-making approaches. Studies should explore whether improved professional competence contributes to more open dialogue, greater disclosure of CAM use, enhanced patient trust and safer integration of patients’ preferences and values into cancer care.

Importantly, the findings should not be interpreted as supporting the routine recommendation of specific CAM interventions. Rather, they highlight the importance of open and respectful communication about CAM use as part of person-centred cancer care. While some complementary approaches may be supported by evidence in specific clinical contexts, the evidence base remains heterogeneous across CAM modalities. The present findings therefore support dialogue, shared decision-making and informed discussions about potential benefits, risks and uncertainties rather than endorsement of CAM interventions per se.

### Practice implications

A pragmatic, safety-oriented and person-centred approach is indicated:

1. Clinicians routinely offer brief, neutral invitations to discuss CAM.
2. Services ensure basic staff competence and signpost reliable evidence resources.
3. Clinics use simple legitimising tools (eg, prompt sheets, brief OD-CAM sessions).
4. Teams address structural barriers—time, documentation and referral pathways.

Together these steps form a practical, multilevel strategy consistent with CAM-communication reviews and the integrative oncology guideline movement.^9 23 25^

### Clinical implications

Open conversations about CAM rarely occur unless patients initiate them, and many hesitate to do so. Clinicians can lower this threshold by signalling, briefly and neutrally, that CAM is welcome to discuss. Basic knowledge of common CAM practices—without implying endorsement—helps staff hold balanced, safety-oriented conversations. Patients valued clear, transparent discussion of possible benefits, uncertainties and limitations to help situate CAM choices within their cancer treatment. Psychosocially, hope, encouragement and being heard mattered. Organisational conditions and evidence norms shaped whether CAM was discussed at all; awareness of these structures can help services create space for dialogue. Recognising CAM as part of many patients’ lived experience supports more coherent, person-centred care.

### Methodological considerations

We used qualitative content analysis with an inductive orientation, moving stepwise from meaning units to codes, categories (manifest level) and themes (latent level). Throughout the analysis, we paid attention to the degree of abstraction and interpretation with a focus on showing how categories and themes were grounded in the study’s aim and logically connected. This approach follows their guidance on working with manifest and latent content, handling inductive-analytic steps and demonstrating credibility in the analytical process.^13 15^

To enhance trustworthiness, we documented key analytic decisions and engaged in peer debriefing on coding, clustering and theme development. We also used reflexive notes and provided thick descriptions to support credibility and dependability. These strategies correspond to established guidance on qualitative content analysis and trustworthiness.^13–15^ While Consolidated criteria for Reporting Qualitative research (COREQ) primarily concerns reporting, several of its items (team/reflexivity, sampling, data collection, analytic transparency) informed how we described methods and context.^32^ The participant quotes box strengthens credibility and dependability by visibly connecting our latent themes to the underlying data, as advocated in qualitative content analysis traditions.^14^

A key reflexive stance in the present analysis was to centre the patients’ voices and avoid causal language in the naming of results—an issue often emphasised in the methodological literature, particularly when working with latent themes. Themes were therefore formulated to capture underlying meaning and process rather than cause-and-effect claims. The same approach guided our handling of statements about system-level factors (eg, evidence norms, regulations, industry influence). We treated these as participants’ experiences and interpretations rather than factual descriptions of the system. This is in line with core principles of qualitative content analysis, which emphasise staying close to the data while allowing interpretation when it is clearly supported.^13 15^

### Strengths and limitations

A strength of the study is the internal consistency of the analytic chain (code → category → theme) and the way results are presented by question, with one main theme per area. This makes the latent thread clear without losing connection to the specific phenomenon of CAM. Methodologically, our approach follows guidance on abstraction, interpretation and analytic transparency.^15^

Limitations include that the data come from one Swedish region and from participants recruited through an earlier survey cohort; transferability should therefore be considered in relation to context. Participants were recruited from both adjuvant oncology and palliative care settings to capture variation in experiences across different phases of the cancer trajectory. However, the study was not designed to compare experiences between these groups, and the analysis focused on patterns across the dataset as a whole. It is therefore possible that differences related to treatment context were not fully captured. Future research may explore whether communication about CAM differs between patients receiving curative and palliative cancer care.

As in many qualitative studies, most of the analysis was conducted by one researcher, supported by peer debriefing rather than full investigator triangulation or member checking. While acceptable, additional strategies could have further strengthened credibility and confirmability. These considerations align with common challenges discussed in qualitative health research, including the role of reflexivity and researcher influence.

## Conclusion

Patients who use CAM alongside cancer treatment navigate a complex care landscape shaped by changing communication patterns, variable openness and structural norms within the healthcare system. They valued encounters that acknowledged their CAM-related decisions and created space to discuss them. Whenever communication was open and responsive, patients described greater trust, confidence and participation; when it was limited or dismissive, they withdrew or avoided disclosure. A care environment that legitimises dialogue about CAM—without implying endorsement—and meets patients with respect, curiosity and informed engagement can strengthen safety and patient-centred care.

## Data Availability

Data are available upon reasonable request.
