# Supplementary material for: Experiences of healthcare encounters among patients using complementary and alternative medicine alongside oncology or palliative care: a qualitative interview study in Sweden
Source: BMJ Open. 2026 Sep 18;16(9):e123561. doi: 10.1136/bmjopen-2026-123561 (PMC13599932; doi:10.1136/bmjopen-2026-123561)
Supplement: Supplementary file 1 [file bmjopen-2026-123561.reviewer_comments.pdf]

PEER REVIEW HISTORY

BMJ Open publishes all reviews undertaken for accepted manuscripts. Reviewers are asked to complete a checklist review form and are provided with free text boxes to elaborate on their assessment. These free text comments are reproduced below.

ARTICLE DETAILS

Title (Provisional)

Experiences of healthcare encounters among patients using complementary and alternative medicine alongside oncology or palliative care: a qualitative interview study in Sweden

Authors

Källman, Mikael; Holgersson, Georg; Bergström, Stefan; Martinsson, Lisa; Lindgren, Britt-Marie; Bergqvist, Michael

---

VERSION 1 - REVIEW

**Reviewer**	**1**
**Name**	**Chang, Hsiao-Ting**
**Affiliation**	**Taipei Veterans General Hospital, Department of Family Medicine**
**Date**	**18-Jul-2026**
**COI**	**None**

Reviewer's comments

Manuscript Title: Experiences of facing healthcare staff among patients who uses Complementary and Alternative medicine alongside oncological or palliative care.

Reviewer's report:

This study aimed to explore how patients experience encounters with healthcare professionals when using CAM alongside oncological or palliative care. This is an interesting study and the authors reported several significant findings from this study. However, I have some recommendations and questions for authors as follows:

Abstract:

1. Please add setting, participants, outcome measures in the Abstract section.

Introduction:

1. What are the hypotheses in this study?

Methods:

1. Please draw a study flow chart for this study.

2. Sampling and participant selection: patients receiving adjuvant chemotherapy or palliative home care are different, please explained the rationale to recruit these patients.

3. Sampling and participant selection: patients received prior chemotherapy administered within the Region Gävleborg healthcare system were excluded, please explain the rationale.

4. Sampling and participant selection: cognitive impairment patients were excluded from this study, please add descriptions for cognitive function assessments.

5. Sampling and participant selection: patients with other comorbidities assessed by the study team as interfering with participation were excluded, please explain the rationale and add descriptions for these comorbidities.

6. Please add details of the interview questionnaire development and contents.

Results:

1. Please add descriptions about characteristic of participants, including how many participants were recruited, their demographic characteristics, and the number of participants who were receiving adjuvant chemotherapy or palliative home care.

2. Are there any different findings from participants who were receiving adjuvant chemotherapy or palliative home care?

Discussion:

1. Patients receiving adjuvant chemotherapy or palliative home care are different, the findings might different for these two groups of patients. Please discussion the potential limitations or provide evidence to support the findings are similar for these two groups of patients.

**Reviewer**	**2**
**Name**	**Fernández-Lázaro, Diego**
**Affiliation**	**Universidad de Valladolid Facultad de Ciencias**
**Date**	**19-Jul-2026**
**COI**	**None**

General comments

The manuscript addresses a relevant and clinically important topic by exploring the experiences of patients with cancer and palliative care needs regarding the use of complementary and alternative medicine (CAM) and their interactions with healthcare professionals. The qualitative design is appropriate for the research question, the methodology is generally rigorous, and the findings provide valuable insights into patient-centered communication and shared decision-making.

The manuscript is well organized, clearly written, and the conclusions are generally supported by the presented data. The topic is timely given the increasing prevalence of CAM use among oncology patients and the need to improve communication between patients and healthcare providers.

Overall, I believe this manuscript represents a valuable contribution to the field and should be considered for publication after minor revision.

Major strengths

Relevant and clinically meaningful research question.

Appropriate qualitative methodology.

Clear presentation of themes and supporting quotations.

Good discussion of communication barriers between patients and healthcare professionals.

Practical implications for oncology and palliative care.

Comments for the authors

1. Expand the Discussion with recent literature

Although the Discussion is comprehensive, it would benefit from incorporating more recent evidence published during 2024–2025 on integrative oncology, supportive care, patient-centered communication, and complementary medicine in cancer survivors.

In particular, the following recent randomized clinical trial may enrich the discussion regarding supportive interventions in breast cancer survivors:

Celorrio San Miguel AM, Cacharro LM, Santamaría G, et al. Adjuvant melatonin therapy during exercise prescription in breast cancer survivors on physical and anthropometric parameters, quality of life, and hormonal response: A randomized controlled trial. Frontiers in Sports and Active Living. 2025;7:1594733.

Although this trial found no additional benefit of melatonin beyond exercise on anthropometric parameters, quality of life, or hormonal profile, it provides an updated perspective on supportive care interventions in breast cancer survivors and illustrates the importance of evidence-based complementary strategies. The findings may help contextualize the current manuscript within the broader framework of integrative oncology.

2. Clinical implications

The Discussion should more clearly distinguish between:

promoting open communication regarding CAM use, and

recommending specific CAM interventions.

Open communication is always desirable, whereas the evidence supporting individual CAM therapies remains heterogeneous.

3. International perspective

The manuscript would benefit from discussing whether the identified communication barriers are specific to the local healthcare system or are consistent with findings reported internationally.

4. Future research

Please include a brief paragraph proposing future research evaluating educational interventions for healthcare professionals aimed at improving communication about CAM and shared decision-making in oncology and palliative care.

5. Minor editorial issues

Only minor language editing is recommended to improve readability in a few sections of the Discussion.

VERSION 1 - AUTHOR RESPONSE

REVIEWER 1

Reviewer's report:
This study aimed to explore how patients experience encounters with healthcare professionals when using CAM alongside oncological or palliative care. This is an interesting study and the authors reported several significant findings from this study. However, I have some recommendations and questions for authors as follows:

We thank the reviewer for the positive and encouraging comments regarding our study. We appreciate the reviewer's careful evaluation of the manuscript and the constructive recommendations provided. We have carefully considered all comments and revised the manuscript accordingly. Detailed point-by-point responses are presented below.

Abstract:
1. Please add setting, participants, outcome measures in the Abstract section.

Response:

Introduction:
1. What are the hypotheses in this study?

Response:

Thank you for this comment. As this study employed an exploratory qualitative design, it was not intended to test predefined hypotheses. Rather, the study was guided by an exploratory research aim focused on understanding patients' experiences of encounters with healthcare professionals regarding CAM use in oncology and palliative care

Methods:
1. Please draw a study flow chart for this study.

Response:

Thank you for this suggestion. To improve clarity regarding participant recruitment and selection, we have expanded the description of the sampling and interview procedures in the Methods section. The revised text now provides a clear account of the survey participants who consented to further contact and the final interview sample included in the qualitative analysis.

2. Sampling and participant selection: patients receiving adjuvant chemotherapy or palliative home care are different, please explained the rationale to recruit these patients.

Response:

Thank you for this comment. Participants were recruited from both oncology and palliative care settings to capture variation in experiences across different phases of the cancer trajectory. While these groups differ regarding treatment goals and clinical context, both represent settings in which CAM use is common and where communication with healthcare professionals about CAM may be relevant. We have clarified this rationale in the Methods section.

3. Sampling and participant selection: patients received prior chemotherapy administered within the Region Gävleborg healthcare system were excluded, please explain the rationale.

Response:

We have clarified that this exclusion criterion originated from the parent survey study from which participants were recruited. In the revised Methods section, we now explain that the present qualitative substudy applied the same eligibility criteria as the parent study

4. Sampling and participant selection: cognitive impairment patients were excluded from this study, please add descriptions for cognitive function assessments.

Response:

Thank you for this comment. No formal cognitive function assessment was conducted. Eligibility was determined through routine clinical evaluation and professional judgement by the clinical staff involved in recruitment. Comorbidities were not predefined but were assessed on a case-by-case basis and could include conditions affecting communication, comprehension, or the ability to provide informed consent and participate in an interview. The exclusion criterion regarding previous chemotherapy within the Region Gävleborg healthcare system was inherited from the parent survey study, and the present qualitative substudy applied the same eligibility criteria. To address this point, we have clarified the eligibility and exclusion criteria in the Methods section while retaining a concise description appropriate for the manuscript.

5. Sampling and participant selection: patients with other comorbidities assessed by the

study team as interfering with participation were excluded, please explain the rationale and add descriptions for these comorbidities.

Response:

Thank you for this commentThe criterion was intended to ensure that participants were able to provide informed consent and meaningfully participate in the interview. No predefined list of comorbidities was used; rather, eligibility was assessed on a case-by-case basis by the clinical staff and research team, taking into account whether a condition could interfere with communication, comprehension, or study participation.

6. Please add details of the interview questionnaire development and contents.

Response:

We have expanded the description of the interview guide development and content in the Data Collection section. The revised text now clarifies that the interview guide was developed from the same domains included in the parent survey study and describes the main topics addressed in the interviews. We also explain that the semi-structured format allowed participants to elaborate on their experiences and enabled the use of follow-up questions to explore issues in greater depth.

Results:

1. Please add descriptions about characteristic of participants, including how many participants were recruited, their demographic characteristics, and the number of participants who were receiving adjuvant chemotherapy or palliative home care.

Response:

We have added a table presenting participant characteristics, including the number of participants included in the study, sex distribution, age, and treatment context. The distribution of participants receiving adjuvant chemotherapy and palliative home care is now reported more clearly.

2. Are there any different findings from participants who were receiving adjuvant chemotherapy or palliative home care?

Response:

Thank you for this comment. Participants from both adjuvant oncology and palliative care settings were included to increase variation in experiences. However, comparison between these groups was not a specific aim of the study, and during this stage of the analysis we focused on themes and patterns across the entire dataset rather than on differences related to treatment context.

Discussion:

1. Patients receiving adjuvant chemotherapy or palliative home care are different, the findings might different for these two groups of patients. Please discussion the potential limitations or provide evidence to support the findings are similar for these two groups of patients.

Response:

Thank you for this important comment. We acknowledge that participants receiving adjuvant chemotherapy and palliative home care represent different clinical contexts and may have distinct experiences. However, comparison between these groups was not a specific aim of the study. Participants from both settings were included to capture variation across different phases of the cancer trajectory, and the analysis focused on patterns across the dataset as a whole. We have added a statement in the Strengths and Limitations section acknowledging that potential differences related to treatment context were not specifically explored and may warrant future investigation.

REVIEWER 2

The manuscript addresses a relevant and clinically important topic by exploring the experiences of patients with cancer and palliative care needs regarding the use of complementary and alternative medicine (CAM) and their interactions with healthcare professionals. The qualitative design is appropriate for the research question, the methodology is generally rigorous, and the findings provide valuable insights into patient-centered communication and shared decision-making.

The manuscript is well organized, clearly written, and the conclusions are generally supported by the presented data. The topic is timely given the increasing prevalence of CAM use among oncology patients and the need to improve communication between patients and healthcare providers.

Overall, I believe this manuscript represents a valuable contribution to the field and should be considered for publication after minor revision.
Major strengths
Relevant and clinically meaningful research question.
Appropriate qualitative methodology.
Clear presentation of themes and supporting quotations.
Good discussion of communication barriers between patients and healthcare professionals.
Practical implications for oncology and palliative care.

We thank the reviewer for these positive comments and for recognising the relevance of the study, the methodological approach, and the potential value of the findings for clinical practice and patient-centred care.

Comments for the authors
1. Expand the Discussion with recent literature

Although the Discussion is comprehensive, it would benefit from incorporating more recent evidence published during 2024–2025 on integrative oncology, supportive care, patient-centered communication, and complementary medicine in cancer survivors.

In particular, the following recent randomized clinical trial may enrich the discussion regarding supportive interventions in breast cancer survivors:

Celorrio San Miguel AM, Cacharro LM, Santamaría G, et al. Adjuvant melatonin therapy during exercise prescription in breast cancer survivors on physical and anthropometric parameters, quality of life, and hormonal response: A randomized controlled trial. Frontiers in Sports and Active Living. 2025;7:1594733.

Although this trial found no additional benefit of melatonin beyond exercise on anthropometric parameters, quality of life, or hormonal profile, it provides an updated perspective on supportive care interventions in breast cancer survivors and illustrates the importance of evidence-based complementary strategies. The findings may help contextualize the current manuscript within the broader framework of integrative oncology.

Response:

Thank you for this valuable suggestion. We agree that incorporating recent literature strengthens the contextualisation of our findings. We have therefore expanded the Discussion by including additional recent research from 2024-2025 within integrative oncology and supportive cancer care. In particular, we have added the suggested randomized controlled trial by Celorrio San Miguel et al. (2025), which illustrates the importance of evaluating complementary interventions using rigorous evidence-based approaches and highlights that not all CAM-related interventions provide additional benefits beyond established supportive care measures. We believe this addition helps situate our findings within the broader and evolving field of integrative oncology.

2. Clinical implications

The Discussion should more clearly distinguish between:

promoting open communication regarding CAM use, and
recommending specific CAM interventions.

Open communication is always desirable, whereas the evidence supporting individual CAM therapies remains heterogeneous.

Response:

Thank you for this important comment. We agree that promoting communication about CAM should be clearly distinguished from recommending specific CAM interventions. We have therefore revised the Discussion to emphasise that our findings support open dialogue, shared decision-making, and informed communication regarding CAM use, rather than endorsement

of particular CAM therapies. We also clarify that the evidence supporting individual CAM modalities remains heterogeneous and should be considered separately from the communication issues explored in this study.

3. International perspective

The manuscript would benefit from discussing whether the identified communication barriers are specific to the local healthcare system or are consistent with findings reported internationally.

Response:

Thank you for this important observation. We agree that it is valuable to consider whether the communication barriers identified in this study are specific to the Swedish healthcare context or reflect broader patterns reported elsewhere. We have therefore expanded the Discussion to compare our findings with international literature. The revised text highlights that limited clinician inquiry about CAM, concerns about disclosing CAM use, and challenges related to discussing CAM within evidence-based clinical frameworks have also been reported in studies from other countries. This suggests that the barriers identified in the present study may reflect broader challenges in oncology and palliative care communication rather than being unique to the local healthcare system.

4. Future research

Please include a brief paragraph proposing future research evaluating educational interventions for healthcare professionals aimed at improving communication about CAM and shared decision-making in oncology and palliative care.

Response:

Thank you for this suggestion. We have added a paragraph to the Discussion emphasising the need for future research evaluating educational interventions for healthcare professionals aimed at improving communication about CAM, shared decision-making, and evidence-informed patient care in oncology and palliative settings.

5. Minor editorial issues

Only minor language editing is recommended to improve readability in a few sections of the Discussion.

Response:

Thank you. We have revised the Discussion section and made minor language edits to improve readability and overall flow.

VERSION 2 - REVIEW

Reviewer 1

**Name**	**Chang, Hsiao-Ting**
**Affiliation**	**Taipei Veterans General Hospital, Department of Family Medicine**
**Date**	**22-Aug-2026**
**COI**	

Thank you for authors' detailed revisions. The manuscript has been substantially improved, and the reviewers' comments have been addressed appropriately.

**Reviewer**	**2**
**Name**	**Fernández-Lázaro, Diego**
**Affiliation**	**Universidad de Valladolid Facultad de Ciencias**
**Date**	**19-Aug-2026**
**COI**	

Thank you for the opportunity to review the revised version of the manuscript entitled "Experiences of facing healthcare staff among patients who uses Complementary and Alternative medicine alongside oncological or palliative care."

The manuscript has improved substantially following revision. The authors have responded carefully to the comments raised during the previous review round and have strengthened several important aspects of the study.

In particular, the revised manuscript now provides clearer information regarding participant recruitment and purposive sampling, inclusion and exclusion criteria, the rationale for including participants from both oncology and palliative care settings, consent procedures, interview-guide development, participant characteristics, and methodological limitations. The qualitative analytic process is also better described, with additional information regarding reflexivity, peer debriefing, analytic decision-making, and trustworthiness.

The structured abstract is clear and appropriately summarizes the setting, participants, main qualitative findings, and clinical implications. The Results are supported by illustrative participant quotations and present a coherent account of how openness regarding CAM varies according to relational cues, professional attitudes, and perceived evidence norms.

I also appreciate the revised Strengths and Limitations section. The authors now explicitly acknowledge that participants receiving adjuvant oncology care and palliative care represent different clinical contexts, that the study was not designed to compare these groups, and that most of the analysis was conducted by one researcher without full investigator triangulation or member checking. These additions improve the transparency and credibility of the manuscript.

The Discussion has also been strengthened through better contextualization with the current integrative oncology literature. The manuscript appropriately emphasizes that open discussion of CAM should not be interpreted as endorsement of CAM interventions, but rather as part of safe, evidence-informed and person-centred communication.

At this stage, I have only a few minor editorial suggestions:

Please revise the title grammatically.

The current wording:

“Experiences of facing healthcare staff among patients who uses Complementary and Alternative medicine alongside oncological or palliative care”

should be grammatically corrected. A clearer option would be:

“Experiences of encounters with healthcare staff among patients using complementary and alternative medicine alongside oncological or palliative care.”

Please conduct one final language-editing pass. A few grammatical or stylistic issues remain, for example expressions such as “Seeking a healthcare that receive CAM…” and isolated agreement/punctuation errors. These do not affect interpretation but should be corrected before publication.

Please ensure consistent terminology and capitalization for complementary and alternative medicine (CAM) throughout the manuscript.

The manuscript appropriately states that no formal cognitive assessment was performed and that eligibility was determined through clinical judgement. I consider this sufficiently transparent; however, the final wording should continue to make clear that cognitive impairment was not assessed using a standardized instrument.

Please ensure that the final proof version contains no residual formatting artefacts from tracked changes or manuscript conversion.

These are minor issues only and do not require additional analysis or substantive revision.

Overall, the revised manuscript presents a relevant and clinically meaningful qualitative study. The research question is important, the methodological approach is appropriate, the themes are well supported by participant quotations, and the implications for communication and patient-centred oncology and palliative care are clear.

Recommendation: Accept after minor editorial revision.

VERSION 2 - AUTHOR RESPONSE

REVIEWER 1

Comments to the Author:

Thank you for authors' detailed revisions. The manuscript has been substantially improved, and the reviewers' comments have been addressed appropriately.

REVIEWER 2

Thank you for the opportunity to review the revised version of the manuscript entitled "Experiences of facing healthcare staff among patients who uses Complementary and Alternative medicine alongside oncological or palliative care."

The manuscript has improved substantially following revision. The authors have responded carefully to the comments raised during the previous review round and have strengthened several important aspects of the study.

In particular, the revised manuscript now provides clearer information regarding participant recruitment and purposive sampling, inclusion and exclusion criteria, the rationale for including participants from both oncology and palliative care settings, consent procedures, interview-guide development, participant characteristics, and methodological limitations. The qualitative analytic process is also better described, with additional information regarding reflexivity, peer debriefing, analytic decision-making, and trustworthiness.

The structured abstract is clear and appropriately summarizes the setting, participants, main qualitative findings, and clinical implications. The Results are supported by illustrative participant quotations and present a coherent account of how openness regarding CAM varies according to relational cues, professional attitudes, and perceived evidence norms.

I also appreciate the revised Strengths and Limitations section. The authors now explicitly acknowledge that participants receiving adjuvant oncology care and palliative care represent different clinical contexts, that the study was not designed to compare these groups, and that most of the analysis was conducted by one researcher without full investigator triangulation or member checking. These additions improve the transparency and credibility of the manuscript.

The Discussion has also been strengthened through better contextualization with the current integrative oncology literature. The manuscript appropriately emphasizes that open discussion of CAM should not be interpreted as endorsement of CAM interventions, but rather as part of safe, evidence-informed and person-centred communication.

At this stage, I have only a few minor editorial suggestions:

Comments for the authors
1. Please revise the title grammatically.

The current wording:

"Experiences of facing healthcare staff among patients who uses Complementary and Alternative medicine alongside oncological or palliative care"

should be grammatically corrected. A clearer option would be:

“Experiences of encounters with healthcare staff among patients using complementary and alternative medicine alongside oncological or palliative care.”

Response: Thank you for this suggestion. As requested by both the reviewer and the editor, the title has been revised for grammatical correctness, clarity, and consistency with BMJ Open's preferred title format. The revised title is:

"Experiences of healthcare encounters among patients using complementary and alternative medicine alongside oncology or palliative care: a qualitative interview study in Sweden."

2. Please conduct one final language-editing pass. A few grammatical or stylistic issues remain, for example expressions such as “Seeking a healthcare that receive CAM…” and isolated agreement/punctuation errors. These do not affect interpretation but should be corrected before publication.

Response: Thank you for this observation. We have conducted a final language-editing review of the entire manuscript and corrected grammatical, stylistic, punctuation, and agreement-related issues throughout the text. In particular, the theme title “Seeking a healthcare that receive CAM with openness, competence, and human presence” has been revised to improve grammatical accuracy and readability. We have also carefully reviewed the manuscript for consistency in language and style before resubmission.

Please ensure consistent terminology and capitalization for complementary and alternative medicine (CAM) throughout the manuscript.

Response: Thank you for this observation. We have reviewed the manuscript and ensured consistent use, terminology, and capitalization of *complementary and alternative medicine (CAM)* throughout the text. We also corrected minor formatting and hyphenation inconsistencies related to CAM terminology where applicable.

The manuscript appropriately states that no formal cognitive assessment was performed and that eligibility was determined through clinical judgement. I consider this sufficiently transparent; however, the final wording should continue to make clear that cognitive impairment was not assessed using a standardized instrument.

Response: Thank you for this observation. To further improve transparency, we have clarified in the Methods section that no formal cognitive assessment was performed and that eligibility regarding cognitive capacity was determined through clinical judgement by healthcare staff involved in the patients’ care

Please ensure that the final proof version contains no residual formatting artefacts from tracked changes or manuscript conversion.

Response: Thank you. We have carefully reviewed the manuscript and removed any residual formatting artefacts. The revised version has been checked to ensure that no tracked changes, comments, or conversion-related formatting issues remain.

These are minor issues only and do not require additional analysis or substantive revision.

Overall, the revised manuscript presents a relevant and clinically meaningful qualitative study. The research question is important, the methodological approach is appropriate, the themes are well supported by participant quotations, and the implications for communication and patient-centred oncology and palliative care are clear.

Recommendation: Accept after minor editorial revision.
